# The Impact of *Staphylococcus aureus*-Associated Molecular Patterns on Staphylococcal Superantigen-Induced Toxic Shock Syndrome and Pneumonia

**DOI:** 10.1155/2014/468285

**Published:** 2014-06-12

**Authors:** Ashenafi Y. Tilahun, Melissa Karau, Alessandro Ballard, Miluka P. Gunaratna, Anusa Thapa, Chella S. David, Robin Patel, Govindarajan Rajagopalan

**Affiliations:** ^1^Department of Immunology, Mayo Clinic, Rochester, MN 55905, USA; ^2^Division of Clinical Microbiology, Department of Laboratory Medicine and Pathology, Mayo Clinic, Rochester, MN 55905, USA; ^3^Division of Infectious Diseases, Mayo Clinic, Rochester, MN 55905, USA; ^4^Department of Immunology and Division of Infectious Diseases, Mayo Clinic College of Medicine, 200 First Street, SW, Rochester, MN 55905, USA

## Abstract

*Staphylococcus aureus* is capable of causing a spectrum of human illnesses. During serious *S. aureus* infections, the staphylococcal pathogen-associated molecular patterns (PAMPs) such as peptidoglycan, lipoteichoic acid, and lipoproteins and even intact *S. aureus*, are believed to act in conjunction with the staphylococcal superantigens (SSAg) to activate the innate and adaptive immune system, respectively, and cause immunopathology. However, recent studies have shown that staphylococcal PAMPs could suppress inflammation by several mechanisms and protect from staphylococcal toxic shock syndrome, a life-threatening systemic disease caused by toxigenic *S. aureus*. Given the contradictory pro- and anti-inflammatory roles of staphylococcal PAMPs, we examined the effects of *S. aureus*-derived molecular patterns on immune responses driven by SSAg *in vivo* using HLA-DR3 and HLA-DQ8 transgenic mice. Our study showed that neither *S. aureus*-derived peptidoglycans (PGN), lipoteichoic acid (LTA), nor heat-killed *Staphylococcus aureus* (HKSA) inhibited SSAg-induced T cell proliferation *in vitro*. They failed to antagonize the immunostimulatory effects of SSAg *in vivo* as determined by their inability to attenuate systemic cytokine/chemokine response and reduce SSAg-induced T cell expansion. These staphylococcal PAMPs also failed to protect HLA-DR3 as well as HLA-DQ8 transgenic mice from either SSAg-induced toxic shock or pneumonia induced by a SSAg-producing strain of *S. aureus*.

## 1. Introduction

*Staphylococcus aureus* is a highly successful opportunistic pathogen. A surprisingly high percentage of healthy humans (up to 40%) can be asymptomatically colonized with* S. aureus* [1]. At the same time,* S. aureus* can cause a wide variety of diseases, from relatively benign skin infections, such as folliculitis and furunculosis, to life-threatening conditions, including erysipelas, deep-seated abscesses, osteomyelitis, pneumonia, endocarditis, sepsis, and toxic shock syndrome (TSS) [2]. Recent epidemiological studies indicate that, at least in the US, the annual mortality caused by the methicillin-resistant strains of* S. aureus* is higher than the mortality caused by HIV/AIDS [3, 4]. Therefore, it is important to understand the immunopathogenesis of invasive diseases caused by* S. aureus*.

The pathogenicity and virulence of invasive* S. aureus* are determined by several exotoxins. Staphylococcal superantigens (SSAg) are one such family of exotoxins produced by* S. aureus*. SSAg are the most potent, naturally occurring biological activators of T lymphocytes [5]. Unlike conventional antigens, SSAg bind directly to cell surface MHC class II molecules outside of the peptide-binding groove without undergoing any processing. Subsequently, they bind to certain T cell receptor *β* chain variable region (TCR V*β*) families and robustly activate the T cells expressing them. The specificity of the SSAg to certain TCR V*β* regions alone, but not the TCR* per se*, results in activation of a large pool of CD4^+^ as well as CD8^+^ T cells (30% to 70% of the total T cells) [5]. SSAg can also activate the antigen presenting cells (APC), such as B cells, monocyte/macrophages, and dendritic cells (DC), by several mechanisms either directly, by crosslinking their MHC class II molecules [6], or indirectly, through cytokines/chemokines. This results in a profound elevation in systemic levels of several cytokines/chemokines which lead to clinical syndromes such as systemic inflammatory response syndrome (SIRS) and multiple organ dysfunction syndrome (MODS), which can culminate in the death of the afflicted patients [7]. As SSAg are produced readily* in vivo*, they play a major role in the pathogenesis of serious infections such as sepsis, pneumonia, and infective endocarditis (reviewed in [8]).

While the SSAg predominantly activate T cells of the adaptive immune system through TCR, certain other staphylococcal components such as the cell wall peptidoglycans, lipoteichoic acid, lipoproteins, unmethylated bacterial DNA containing the CpG motifs and even the intact bacteria, collectively called pathogen-associated molecular patterns (PAMPs), can activate the immune system through pathogen recognition receptors (PRR) comprising the toll-like receptors (TLR) and the NOD-like receptors (NLR), expressed by cells of both innate and adaptive arms of the immune system [9–11]. This leads to production of several proinflammatory cytokines, chemokines, and other inflammatory mediators [6, 12–16]. Some studies have shown that staphylococcal PAMPs can even directly activate T cells to a proinflammatory phenotype in a TLR2-Myd88 dependent manner [17], while others have demonstrated that* S. aureus* and staphylococcal PAMPs are able to override the immune regulatory functions of T regulatory cells, thereby indirectly promoting inflammation [18]. Overall, staphylococcal PAMPs can also promote inflammation and immunopathology similar to SSAg by several different mechanisms.

Given that both the SSAg and the staphylococcal PAMPs elicit a predominantly proinflammatory type of immune response [16, 19, 20], it is widely believed that, during invasive* S. aureus* infections, the PAMPs would act additively or synergistically with SSAg leading to a more pronounced inflammatory response and inflicting severe immunopathology [7, 21]. On the contrary, some recent studies have shown that the staphylococcal cell wall components and certain other staphylococcal PAMPs downregulate the immune response to SSAg as well as dampen antistaphylococcal immunity [22–24]. Thus, the staphylococcal PAMPs appear to have opposing immunological properties according to different studies. Given the significance of* S. aureus* infections [2, 25], it is important to understand the interaction between staphylococcal PAMPs and SSAg* in vivo* using appropriate animal models. As transgenic mice expressing human MHC class II molecules closely mimic the human immune response to infections caused by toxigenic staphylococci as well as streptococci producing superantigens [26–29], we examined the modulatory role of staphylococcal PAMPs in immune response to SSAg and the outcome of* S. aureus* pneumonia using HLA-DR3 and HLA-DQ8 transgenic mice.

## 2. Materials and Methods

### 2.1. Mice

HLA-DR3 transgenic mice expressing HLA-DRA1∗0101 and HLA-DRB1∗0301 transgenes and HLA-DQ8 transgenic mice expressing DQA1∗0301 and DQB1∗0302 on the endogenous MHC class II-null background were used in this study [30, 31]. Hereafter, they are referred to as HLA-DR3 or HLA-DQ8 mice, respectively. Mice were bred within the barrier facility of Mayo Clinic Immunogenetics Mouse Colony (Rochester, MN) and moved to a conventional facility after weaning. All experiments were approved by the Institutional Animal Care and Use Committee.

### 2.2. Antibodies and Reagents

The following antibodies were used for flow cytometry: CD4 (clone: GK1.5), CD8 (clone: 53–6.7), TCR V*β*6 (clone: RR4-7), TCR V*β*8 (clone: F23.1), CD86 (clone: GL-1), and class II (clone: Tu39). All antibodies were from BD Biosciences (San Jose, CA). Highly purified, endotoxin-reduced, staphylococcal enterotoxin B (SEB, Toxin Laboratories, Sarasota, FL, USA) was dissolved in PBS at 1 mg/mL and stored frozen at −80°C in aliquots. Peptidoglycans (PGN) purified from* S. aureus*, lipoteichoic acid (LTA) derived from* S. aureus*, and heat-killed* Staphylococcus aureus* (HKSA) were purchased from Invivogen (San Diego, CA), dissolved or resuspended in endotoxin-free PBS, respectively, and stored frozen at −80°C. The above-mentioned TLR agonists from this vendor have been extensively used in biomedical research.

### 2.3. *In Vitro* Cultures

For T cell proliferation, single-cell suspensions of splenocytes from HLA-DR3 and HLA-DQ8 transgenic mice were depleted of red blood cells by buffered ammonium chloride lysis. Cells were cultured in HEPES-buffered RPMI 1640 containing 5% fetal calf serum, serum supplement, streptomycin, and penicillin, at a concentration of 1 × 10^5^ cells/well in 100 *μ*L volumes in 96-well round-bottomed tissue culture plates. SEB, PGN, and HKSA were added at indicated concentrations at the same time. After 24 hours, the cells were pulsed with tritiated thymidine (1 *μ*g/well). Cells were harvested 18 hours later and the extent of cell proliferation was determined by a standard thymidine incorporation assay.

To study the effect of staphylococcal PAMPs on expression of MHC class II molecules and costimulatory molecules, splenocytes from HLA-DR3 transgenic mice were cultured with medium alone or with HKSA (10^8^ bacteria/mL), SEB (1 *μ*g/mL), or SEB + HKSA (10^8^ bacteria/mL + 1 *μ*g/mL, resp.) in 24-well plates. Twenty-four hours later, the cells were obtained, washed, stained with indicated antibodies, and analyzed by flow cytometry. Flow data were analyzed using FlowJo X V10.0.6. Mononuclear cells were first gated based on forward and side scatter profiles. Other analyses were performed on cells within this gate.

### 2.4. *In Vivo* Studies

For serum cytokine analyses, HLA-DR3 mice were challenged with SEB (50 *μ*g), PGN (50 *μ*g), HKSA (10^8^ bacteria), or SEB plus PGN or HKSA at these concentrations. The doses of these reagents were determined from previously published studies [22, 32–34]. Given the rapidity at which SSAg cause immune activation, these staphylococcal PAMPs were administered immediately after SEB. After 3 hrs, blood was collected in serum separation tubes (BD Biosciences), and sera were separated and stored frozen at −80°C in aliquots. The cytokine concentrations in the sera were determined in duplicate using a multiplex bead assay, per the manufacturer's protocol, and using their software and hardware (Bio-Plex and Bio-Rad).

For flow cytometric studies, HLA-DR3 mice were challenged with SEB (10 *μ*g), PGN (50 *μ*g), LTA (50 *μ*g), or SEB plus PGN or LTA at the above indicated concentrations. Mice were killed 3 days later and distribution of CD4^+^ and CD8^+^ T cells bearing TCR V*β*8 and V*β*6 in the spleens was determined by flow cytometry as per standard protocol.

To study the modulatory role of staphylococcal PAMPs in SEB-induced mortality without D-gal sensitization, HLA-DR3 mice were challenged with SEB (50 *μ*g), PGN (50 *μ*g), HKSA (10^8^ bacteria), or SEB plus PGN or HKSA. Animals were monitored closely and the moribund animals were euthanized as per IACUC recommendations. To study the modulatory role of staphylococcal PAMPs in SEB-induced mortality with D-gal sensitization, HLA-DR3 and HLA-DQ8 transgenic mice were pretreated with D-gal (30 mg/mouse). One hour later, animals were challenged with SEB (5–10 *μ*g), PGN (50 *μ*g), HKSA (10^8^ bacteria), or SEB plus PGN or HKSA.

### 2.5. Induction of Pneumonia with Toxigenic* S. aureus*

We have recently demonstrated that HLA-DR3 transgenic mice are susceptible to pneumonia induced by a toxigenic strain of* S. aureus*, IDRL-7971, producing the SSAg, SEA, and SEB as tested by PCR and ELISA [29, 35]. Therefore, to study the modulatory role of staphylococcal PAMPs in pneumonia induced by a superantigen-producing strain of* S. aureus*, HLA-DR3 transgenic mice were intratracheally challenged as described previously with the* S. aureus* strain, IDRL-7971 (1.3–2.5 × 10^8^ cfu/mouse). Immediately following bacterial inoculation, mice were left untreated or injected with HKSA intraperitoneally (10^8^ bacteria/mouse). Animals were monitored closely for symptoms and moribund animals were removed from the study.

### 2.6. Statistics

Statistical analyses, charts, and generation of survival curves were done using GraphPad Prism, version 4.03.

## 3. Results 

### 3.1. Modulation of HLA Class II Expression by Staphylococcal PAMPs

Direct binding to cell surface MHC class II molecules without undergoing processing is the first step in the series of events by which SSAg causes immune activation [5]. Therefore, any alterations in the cell surface expression of MHC class II molecules on APCs could modulate the response elicited by SSAg. In this context, some studies have shown that staphylococcal PAMPs upregulate the expression of MHC class II as well as costimulatory molecules on APCs [36], while others have shown that staphylococcal PAMPs rather downregulate the expression of MHC class II molecules, particularly on monocytes [22–24]. Since many types of APCs are involved in the presentation of SSAg in addition to monocytes, we investigated the effect of staphylococcal PAMPs on the expression of MHC class II molecules on different APCs. As HKSA would encompass all staphylococcal PAMPs, splenocytes from HLA-DR3 transgenic mice were cultured with HKSA or SEB or both and the abilities of these agents to modulate the cell surface expression of HLA-DR3 and CD86 on B220^+^ cells (predominantly B cells), CD11b^+^ cells (predominantly monocyte/macrophages), and CD11c^+^ cells (predominantly DC) were determined.

**Figure 1:**
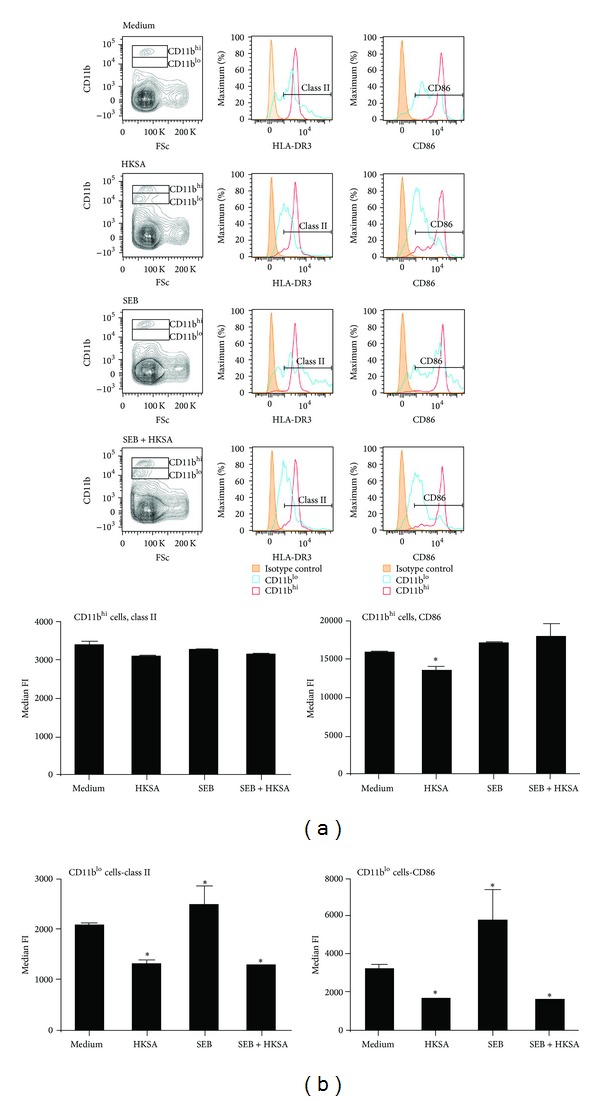
Modulation of expression of HLA-DR3 and CD86 on CD11b^+^ cells by HKSA. Splenic mononuclear cells from HLA-DR3 transgenic mice were cultured with medium, HKSA (10^8^ bacteria/mL), SEB (1 *μ*g/mL), or SEB + HKSA. 24 hours later, the cells were harvested and washed and expression of HLA-DR3 and CD86 on CD11b^+^ cells was analyzed by flow cytometry. Mononuclear cells were first gated based on forward and side scatter profiles. Other analyses were performed on cells within this gate. Representative histogram profiles and bar charts depicting median fluorescent intensity (MFI) are given. Each bar represents mean ± SE from 2 different experiments, each performed in triplicate. *P < 0.05 compared to cells cultured with medium.

We first studied the effects of HKSA on MHC class II expression on CD11b^+^ splenocytes. Interestingly, unlike splenocytes cultured with medium alone, two distinct subsets of CD11b^+^ cells could be appreciated in splenocytes cultured with HKSA based on the expression profile of CD11b, with one expressing high levels of CD11b (CD11b^hi^) and the other expressing lower levels, CD11b^lo^ (Figure 1). In cells cultured with medium alone, CD11b^hi^ was the dominant phenotype (nearly 80–90% of the CD11b^+^ cells were of the CD11b^hi^ phenotype). However, in cells cultured with HKSA, 50–60% of the CD11b^+^ cells were of the CD11b^lo^ phenotype. In a similar manner, while 80–90% of the CD11b^+^ cells cultured with SEB were CD11b^hi^, >50% of the CD11b^+^ cells were CD11b^lo^ when cultured with SEB + HKSA (Figure 1). Therefore, we studied the expression profiles of HLA-DR3 and CD86 on CD11b^hi^ as well as CD11b^lo^ subsets. HKSA had very little suppressive effect on the expression of HLA-DR3 within the CD11b^hi^ subset either when cultured alone or with SEB (Figure 1). However, the expression of HLA-DR3 was significantly reduced in the CD11b^lo^ subsets that were generated in the presence of HKSA either alone or along with SEB. Similar pattern was seen with respect to expression of CD86. Overall, HKSA lowered the expression of HLA-DR3 and CD86 only on a subset of CD11b^+^ cells (Figure 1).

**Figure 2:**
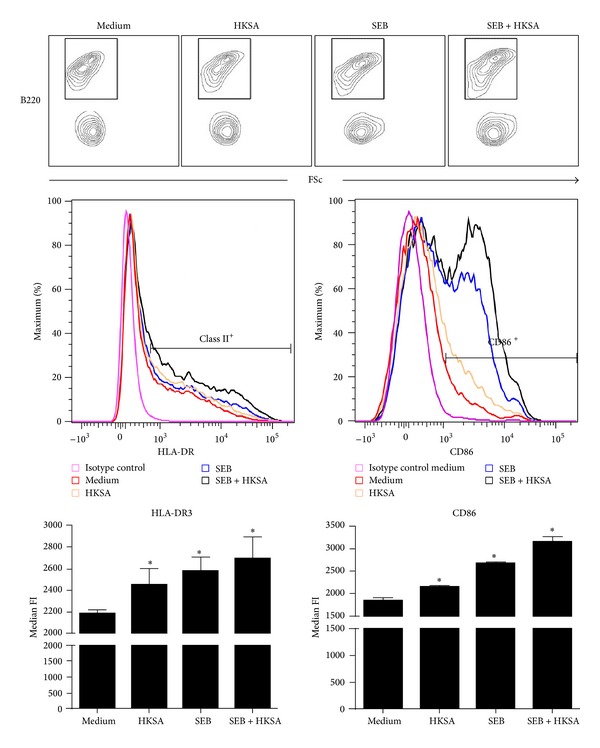
Modulation of expression of HLA-DR3 and CD86 on B220^+^ cells by HKSA. Splenic mononuclear cells from HLA-DR3 transgenic mice were cultured with medium, HKSA (10^8^ bacteria/mL), SEB (1 *μ*g/mL), or SEB + HKSA. 24 hours later, the cells were harvested and washed and expression of HLA-DR3 and CD86 on B220^+^ cells was analyzed by flow cytometry. Mononuclear cells were first gated based on forward and side scatter profiles. Other analyses were performed on cells within this gate. Representative histogram profiles and bar charts depicting median fluorescent intensity (MFI) are given. Each bar represents mean ± SE from 2 different experiments, each performed in triplicate. *P < 0.05 compared to cells cultured with medium.

We next studied the effect of HKSA on the expression of HLA-DR3 and CD86 on B220^+^ splenocytes. Unlike in CD11b^+^ cells, culturing with HKSA did not skew the B220^+^ cells to either B220^hi^ or B220^lo^ phenotype (Figure 2). Therefore, the entire B220^+^ population was studied. Unlike in CD11b^+^ cells, HKSA significantly increased the expression of HLA-DR3 molecules on B220^+^ cells over the basal level (median fluorescent intensity or MFI; 2185 ± 36 versus 2451 ± 151 in medium versus HKSA treated cells, respectively; *P* < 0.05; Figure 2). Culturing with SEB also significantly increased the expression of HLA-DR3 molecules on B220^+^ cells (MFI in SEB treated cells, 2581 ± 127 and *P* < 0.05, compared to untreated cells). Notably, SEB induced a much stronger upregulation of HLA-DR3 than HKSA. However, HKSA failed to suppress SEB-induced upregulation of HLA-DR3 on B220^+^ cells. On the contrary, expression of HLA-DR3 further increased when cultured with SEB + HKSA (MFI in SEB + HKSA treated cells, 2695 ± 205 and *P* < 0.05, compared to medium) (Figure 2). Similar pattern was noticed with respect to CD86 expression on B220^+^ cells. Culturing with HKSA caused a significant (*P* < 0.05) upregulation of CD86 levels on B220^+^ cells compared to untreated cells, while SEB caused a much stronger upregulation of CD86 compared to HKSA. However, culturing with HKSA and SEB resulted in the highest upregulation of CD86 compared to either of these agents alone. These results indicated that HKSA failed to suppress SEB-induced upregulation of HLA-DR3 and CD86 on B220^+^ cells. Similar phenomenon was seen in CD11c^+^ cells. HKSA, SEB, and their combination increased the expression of HLA-DR3 and CD86 expression on CD11c^+^ cells (see Supplementary Figure 1 in Supplementary Material available online at http://dx.doi.org/10.1155/2014/468285). Overall, these results suggested that HKSA augmented the expression of MHC class II and CD86 on B220^+^ cells and DC. While HKSA had minimal or no modulatory effect on CD11b^hi^ cells in the presence of SEB, it reduced the expression of MHC class II and CD86 on the CD11b^lo^ cells, which were seen in the presence of HKSA.

### 3.2. Modulation of SSAg-Induced T Cell Activation by Staphylococcal PAMPs* In Vitro*

**Figure 3:**
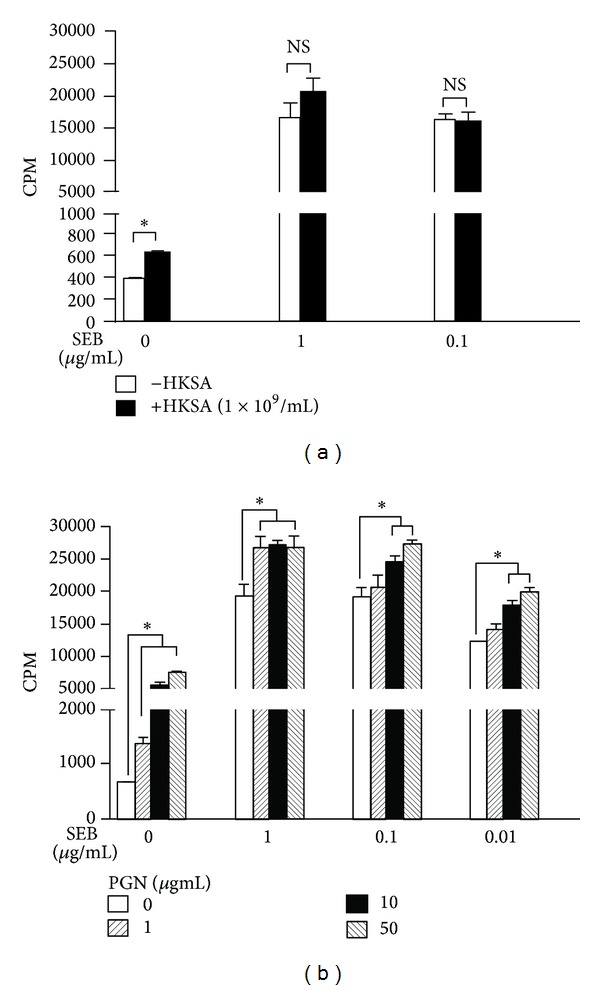
Effect of* S. aureus*-derived PAMPs on SEB-induced T cell proliferation* in vitro*. Splenic mononuclear cells from HLA-DR3 transgenic mice were cultured with indicated concentrations of SEB in the presence or absence of HKSA, 10^8^ bacteria/mL (a), or PGN (b). T cell proliferation was determined by thymidine incorporation. *P < 0.05 compared to cells cultured without HKSA or PGN within that group. Each bar represents mean ± SE from triplicate wells. Representative data from one out of 3 similar experiments are given.

We next investigated how modulation in the expression of MHC class II molecules and CD86 induced by staphylococcal PAMPs translated into changes in SSAg-mediated T cell activation* in vitro*. For this, splenocytes from HLA-DR3 transgenic mice were stimulated with SEB in the presence or absence of certain staphylococcal PAMPs. First of all, HKSA by itself was weekly mitogenic to splenocytes. Most importantly, HKSA failed to suppress SEB-induced splenocyte proliferation* in vitro* (Figure 3(a)). We next tested the modulatory effects of PGN. PGN by itself was significantly mitogenic to splenocytes from HLA-DR3 mice (Stimulation Index ranging from 2.5 to 12 depending on the dose of PGN; *P* < 0.05; Student's *t*-test; Figure 3(b)). However, as would be expected of a superantigen, SEB was more mitogenic to splenocytes than PGN. More importantly, addition of PGN did not suppress SEB-induced splenocyte proliferation. Conversely, PGN, especially at 10 and 50 *μ*g/mL, consistently augmented SEB-induced splenocyte proliferation (*P* < 0.05; Student's *t*-test). Similar results were obtained with HLA-DQ8 transgenic mice using PGN and HKSA (data not shown). Overall, PGN and HKSA failed to suppress SEB-induced splenocyte proliferation* in vitro*. On the contrary, PGN augmented SEB-induced splenocyte proliferation at higher concentration.

It should be noted that SSAg cause a systemic inflammatory disease characterized by failure of several vital organs that often leads to death [37], and* in vitro* studies with isolated splenocytes reflect very little about this systemic process. Therefore, to accurately determine the modulatory role of staphylococcal PAMPs* in vivo*, we subsequently performed a series of* in vivo* studies using our robust HLA-DR3 and HLA-DQ8 transgenic mice, which closely mimic the human immune responses to SSAg [38].

### 3.3. Modulation of SSAg-Induced Cytokine/Chemokine Elevation by Staphylococcal PAMPs* In Vivo*

**Figure 4:**
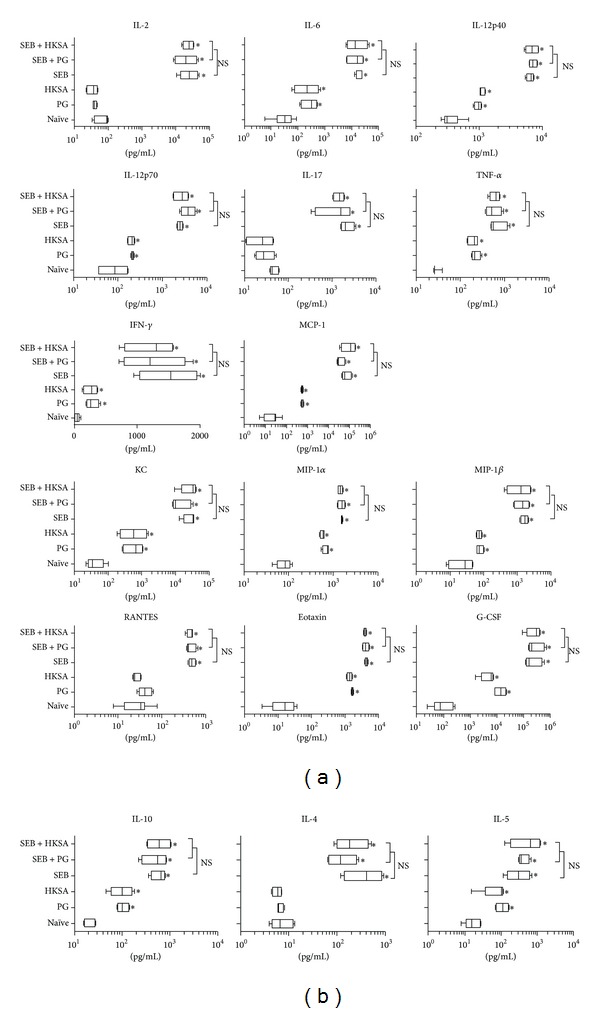
Effect of* S. aureus*-derived PAMPs on SEB-induced systemic inflammatory response syndrome* in vivo*. HLA-DR3 transgenic mice were challenged with staphylococcal enterotoxin B (SEB) alone (50 *μ*g), staphylococcal peptidoglycan (PGN) alone (50 *μ*g), or heat-killed* Staphylococcus aureus* (HKSA) alone (10^8^ bacteria), SEB with PGN or SEB with HKSA. Animals bled 4 hours after injection and serum cytokines were quantified using Bioplex assays (Bio-Rad). Mean ± SE from 4–6 mice in each group. *P < 0.05 compared to naïve mice.

Challenging HLA-DR3 transgenic mice with SSAg, such as SEB, elicits a massive and rapid elevation in systemic levels of various cytokines and chemokines leading to SIRS, multiple organ failure, and death, analogous to TSS in humans [38]. Therefore, we next investigated the modulatory effect of staphylococcal PAMPs on SEB-induced systemic inflammatory response syndrome. Serum cytokine analysis showed that naïve HLA-DR3 mice had very low levels of cytokines/chemokines (Figure 4). As expected, the serum levels of several cytokines (such as IL-6, IL12p40, IL-12p70, IL-10, IFN-*γ*, and TNF-*α*) and chemokines (MCP-1, KC, MIP-1*α*, MIP-1*β*, eotaxin, and G-CSF) were significantly elevated in mice challenged with PGN or HKSA compared to naïve mice, likely resulting from activation of the innate immune system. Given the biological property of SEB, it is not surprising to find that sera from HLA-DR3 mice challenged with SEB had profoundly elevated levels of all the cytokines and chemokines tested (Figure 4), consistent with our prior reports [30]. Moreover, the serum levels of all cytokines and chemokines tested were significantly higher in SEB treated mice compared to PGN or HKSA treated mice. This is also expected because SSAg are the most potent biological activators of the immune system. More importantly, when PGN and HKSA were injected along with SEB, they failed to suppress SEB-induced cytokine/chemokine production. There were no significant differences in the levels of various cytokines and chemokines tested between mice challenged with SEB alone compared to SEB + PGN or SEB + HKSA. Previous studies have shown that staphylococcal cell wall derivatives are more potent inducers of IL-10 than SSAg [22]. On the contrary, our studies show that SEB is more potent than staphylococcal PAMPs in eliciting IL-10. Overall, our results show that the staphylococcal PAMPs do elicit an immune response. However, they are less potent than SSAg and they failed to attenuate SEB-induced cytokine production* in vivo*.

### 3.4. Modulation of SSAg-Induced Peripheral T Cell Expansion by Staphylococcal PAMPs* In Vivo*

**Figure 5:**
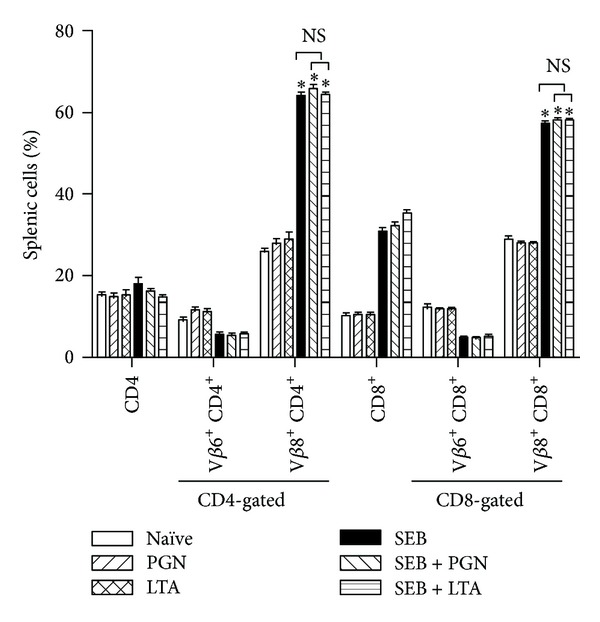
Effect of* S. aureus*-derived PAMPs on SEB-induced T cell expansion* in vivo*. HLA-DR3 transgenic mice were challenged with staphylococcal enterotoxin B (SEB) alone (10 *μ*g), staphylococcal peptidoglycan (PGN) alone (50 *μ*g), or lipoteichoic acid (LTA, 50 *μ*g), SEB with PGN or SEB with LTA. Animals were killed 3 days later and distribution of T cells expressing TCR V*β* families were determined by flow cytometry. Mean ± SE from 5–8 mice in each group. *P < 0.05 compared to naïve mice. *p* = NS between SEB versus SEB + PGN, SEB versus SEB + LTA, and SEB + LTA versus SEB + PGN.

Administration of SSAg, such as SEB, into HLA class II transgenic mice results in the expansion of CD4^+^ as well as CD8^+^ T cells expressing certain TCR V*β* families. It is known that this process is dependent on expression of MHC class II molecules and is cytokine driven. Therefore, by comparing the changes in T cell repertoire in mice challenged with SEB alone or SEB along with staphylococcal PAMPs, we were able to study the immunomodulatory functions of staphylococcal PAMPs. Compared to naïve mice, there was a 2-fold increase of CD4^+^ and CD8^+^ T cells expressing TCR V*β*8, which specifically binds to SEB, in mice challenged with SEB (Figure 5). As expected, PGN and LTA did not have this ability to induce expansion of T cells bearing specific TCR V*β*. More importantly, neither PGN nor LTA was able to suppress SEB-induced expansion of TCR V*β*8-bearing T cells (Figure 5; *p* = NS when comparing SEB with SEB + PGN or SEB with SEB + LTA). This corroborated with the* in vivo* cytokine data.

### 3.5. Modulation of SSAg-Induced TSS by Staphylococcal PAMPs

**Figure 6:**
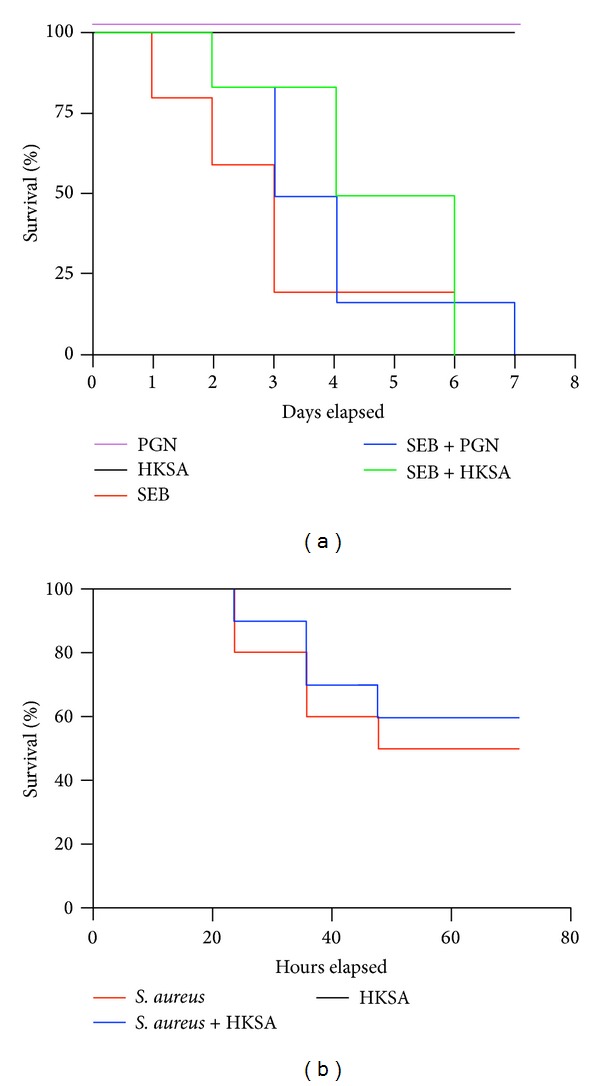
Effect of* S. aureus*-derived PAMPs on SEB-induced TSS and* S. aureus*-induced pneumonia. (a) HLA-DR3 transgenic mice were challenged with staphylococcal enterotoxin B (SEB) (50 *μ*g), staphylococcal peptidoglycan (PGN) (50 *μ*g), or heat-killed* Staphylococcus aureus* (HKSA) (10^8^ bacteria), SEB with PGN or SEB with HKSA. Animals were monitored every 6 hours (4–8 mice per group). (b) HLA-DR3 transgenic mice were challenged intratracheally with a toxigenic strain of* S. aureus* capable of producing the SSAg, SEA, and SEB (1.3–2.5 × 10^8^ cfu/mouse). Immediately following bacterial inoculation, mice were left untreated or injected with HKSA intraperitoneally (10^8^ bacteria/mouse). Animals were monitored closely for symptoms. All moribund animals were removed from the study (6–8 mice per group).

We next investigated the ability of staphylococcal PAMPs to protect from SSAg-induced TSS and death. Whereas all mice challenged with PGN or HKSA alone remained healthy, all HLA-DR3 mice challenged with SEB (50 *μ*g) alone either succumbed to TSS or became very sick that they needed to be removed from the study (*P* < 0.005; the Log-rank (Mantel-Cox) test; Figure 6(a); 4–8 mice in each group). Interestingly, unlike in the previous report [22], administration of either PGN or HKSA failed to protect HLA-DR3 transgenic mice from SEB-induced TSS. Even though mortality in SEB + HKSA treated mice was delayed initially compared to SEB or SEB + PGN groups, all mice in this group succumbed earlier to TSS compared to other groups. Overall, there were no statistical differences in the survival between SEB, SEB + PGN, and SEB + HKSA groups (*p* = NS; the Log-rank (Mantel-Cox) test). However, the survival of all SEB-challenged mice (SEB alone, SEB + PGN, or SEB + HKSA) was significantly reduced compared to naïve and PGN or HKSA challenged mice (*P* < 0.005; Figure 6(a); the Log-rank (Mantel-Cox) test).

It should be noted that the above experiments were done without D-gal sensitization. As conventional laboratory mouse strains are resistant to SSAg-induced shock, they are often presensitized with D-gal. D-gal is a hepatotoxic agent and sensitizes hepatocytes to TNF-*α*-mediated cell death [32, 39]. Therefore, in the next set of experiments, we pretreated HLA-DR3 mice with D-gal and one hour later mice were challenged with SEB (5–10 *μ*g), PGN (50 *μ*g), HKSA (10^8^ bacteria), SEB plus PGN, or SEB plus HKSA, similar to the study by Chau et al. [22]. While all mice treated with D-gal alone, D-gal plus PGN, or D-gal plus HKSA remained healthy, all animals treated with D-gal and SEB (either alone or along with PGN or HKSA) rapidly became hypothermic and lethargic and failed to survive beyond 9 hours (6–8 mice in each group; Table 1). There were no significant differences in survival between D-gal plus SEB, D-gal plus SEB plus PGN, and D-gal plus SEB plus HKSA groups. To rule out the possibility that the lack of protection from TSS by PGN and HKSA may be specific for HLA-DR3, we repeated this study with HLA-DQ8 transgenic mice. HLA-DQ8 transgenic mice were pretreated with D-gal as described earlier and subsequently challenged with SEB (10 *μ*g), PGN (10 or 50 *μ*g), HKSA (10^8^ bacteria), SEB plus PGN, or SEB plus HKSA. As seen with HLA-DR3 transgenic mice, PGN and HKSA failed to protect from SEB-induced TSS in HLA-DQ8 transgenic mice (4–6 mice in each group; Table 1). Overall, staphylococcal PAMPs conferred little protection from SSAg-induced TSS.

### 3.6. Role of Staphylococcal PAMPS in Outcome of* S. aureus*-Induced Lethal Pneumonia

In the final* in vivo* study, we investigated the immunomodulatory effects of staphylococcal PAMPs on pneumonia induced superantigen-producing* S. aureus* [29, 35]. Since HKSA would encompass all the staphylococcal PAMPs, infected animals were treated with HKSA. As expected, HKSA by itself was not lethal (Figure 6(b)). As shown by us earlier, there was significantly higher mortality (*P* < 0.01; the Log-rank (Mantel-Cox) test) in HLA-DR3 mice infected with the toxigenic* S. aureus* strain IDRL-7971 compared to mice treated with HKSA alone [29, 35]. Moreover, the mortality remained significantly high in HLA-DR3 transgenic mice that were infected with IDRL-7971 and treated with HKSA. The survival curves of mice with IDRL-7971-induced pneumonia and those with IDRL-7971-induced pneumonia treated with HKSA were not significantly different (*P* = 0.62; the Log-rank (Mantel-Cox) test). Overall, these results suggested that staphylococcal PAMPs do not confer significant protection in certain staphylococcal diseases such as TSS and pneumonia.

## 4. Discussion

The pattern recognition receptors (PRR) expressed by the cells of the innate immune system rapidly sense PAMPs [10] and mount an inflammatory response to contain infection [40, 41]. PAMPs associated with* S. aureus* are no exceptions [9, 20, 42]. However, the superantigen exotoxins produced by* S. aureus* (SSAg) predominantly activate the T cell arm of adaptive immunity by directly binding to MHC class II molecules and subsequently stimulating the T lymphocytes expressing certain TCR V*β* families [5]. Given that both staphylococcal PAMPs and SSAg activate the immune system, exposure to both of these staphylococcal entities, as might occur in many clinical conditions such as staphylococcal sepsis, pneumonia, or toxic shock syndrome (TSS), is believed to result in synergistic activation of the innate and adaptive immune system and a worse disease outcome [7, 21, 43]. However, some recent studies have suggested that staphylococcal PAMPs may actually suppress the immune response to SSAg and cause immune tolerance or even immune deviation following* S. aureus* infections [22–24]. Given these conflicting reports, we investigated the immunomodulatory role of staphylococcal PAMPs in immune responses elicited by SSAg during TSS and in staphylococcal pneumonia. Our studies, which used the robust HLA class II transgenic mouse model, showed that staphylococcal PAMPs do not suppress SSAg-induced T cell proliferation, do not dampen SSAg-induced systemic inflammatory response, and fail to protect from SSAg-induced TSS or staphylococcal pneumonia.

One of the major hypotheses put forth by the previous* in vitro* studies addressing the immunomodulatory properties of staphylococcal PAMPs is that they cause immunomodulation by downregulating the expression of MHC class II on macrophages [22, 24]. These studies demonstrated that the staphylococcal cell wall components bind to TLR2, which are constitutively expressed on monocyte/macrophages and downregulate their MHC class II molecule expression [22, 24]. This diminishes the ability of monocyte/macrophages to present SSAg, causing reduced T cell activation, immunosuppression/immune deviation, and protection from TSS [22, 24]. However, these studies did not take into consideration the role of other APCs that are present* in vivo*.

It is well known that, in addition to monocytes, HLA class II molecules are constitutively expressed at very high levels on several APC types, including DC and B cells. Particularly, B cells express high levels of MHC class II molecules and can efficiently present SSAg. While we were able to reproduce the findings of Frodermann et al. and Wang et al. that HKSA could downregulate the expression of MHC class II on a subset of CD11b^+^ cells [23, 24], surprisingly HKSA had little or no suppressive effect on the expression of MHC class II molecules on B220^+^ cells (Figures 1 and 2) and CD11c^+^ (Supplementary Figure 1). Rather HKSA augmented the expression of MHC class II as well as CD86, an important costimulatory molecule on these APC subsets. Since B cells and DC vastly outnumber the monocyte/macrophages in the spleen, it is not surprising that staphylococcal PAMPs had no net immunosuppressive effect* in vitro* and* in vivo*. The discrepancy between previous studies and our study could be attributed to the use of human cells versus cells from HLA class II transgenic mice, respectively.

In addition to professional APC, even human T cells are known to express HLA class II molecules upon activation. In a similar manner, in addition to the conventional APCs, MHC class II (HLA-DR3) molecules are also expressed on activated T cells in our transgenic mice, as shown by RT-PCR and flow cytometry. T cells from HLA-DR3 transgenic mice can present antigens and SSAg to other T cells [44]. Thus, HLA class II molecules are widely expressed by several professional and nonprofessional APCs. Given the abundance of HLA class II molecules* in vivo*, the affinities of SSAg for HLA class II molecules, the rapidity (SSAg can elicit an immune response within minutes) and robustness with which SSAg elicit an immune response, and the presentation of SSAg by professional and nonprofessional APCs (including T lymphocytes) might rapidly activate the immune system and override the regulatory effects of TLR2. Our* in vitro* and* in vivo* experiments support this hypothesis.

Another mechanism by which staphylococcal PAMPs mediate immune modulation is through induction of IL-10, specifically by the monocytes [23, 24]. However, staphylococcal PAMP-driven, IL-10-mediated immunomodulation has been shown to be abolished by INF-*γ* [23, 24]. We and others have consistently shown that SSAg readily induce IFN-*γ* and systemic levels of IFN-*γ* are elevated (both IFN-*γ* mRNA and protein) in mice (even in the current study) and humans undergoing TSS [27, 32, 38]. Therefore, while staphylococcal PAMPs could suppress the immune response through monocyte/macrophage-derived IL-10, elevated levels of proinflammatory cytokines (such as IL-12, IFN-*γ*, and IL-17) might overcome this suppressive effect. In this context, other studies have shown that even human PBMCs stimulated with SSAg and exposed to staphylococcal PAMPs or HKSA produced significantly higher levels of proinflammatory cytokines [45]. Even staphylococcal PAMPs by themselves can induce the production of proinflammatory cytokines by human PBMC and promote immunopathology [34, 46–48].

Finally, the animal model used to investigate the interaction between staphylococcal PAMPs and SSAg also plays a major role in drawing apt conclusions. We have shown that our HLA class II transgenic mice respond robustly to SSAg and are highly susceptible to* S. aureus*-induced pneumonia as well as SSAg-induced TSS without requiring any sensitizing agents such as LPS or D-galactosamine [38]. Therefore, the heightened potency of SSAg in HLA class II transgenic mice (and in humans) may be beyond the realm of immunoregulation by staphylococcal PAMPs. Taken together, our results suggest that staphylococcal PAMPs offer little protection during staphylococcal TSS and pneumonia conditions wherein higher amounts of SSAg are present. High mortality rates associated with invasive* S. aureus* infections underscore the fact that even in humans staphylococcal PAMPs are unable to effectively mitigate SIRS and MODS. However, as suggested in a recent study [49], it is possible that staphylococcal PAMPs might play an immunosuppressive or immune deviatory role during* S. aureus* colonization where elevated levels of SSAg are not expected to be present. This needs further investigation.

## Supplementary Material

Splenic mononuclear cells from HLA-DR3 transgenic mice were cultured with medium, HKSA (108 bacteria/ml), SEB (1*μ*g/ml) or SEB*+*HKSA. 24 hours later, the cells were harvested, washed and expression of HLA-DR3 and CD86 on CD11c*+* was analyzed by flow cytometry. Charts depict mean fluorescent intensity (MFI). Each bar represents mean ± SE from 2 different experiments.

## Figures and Tables

**Table 1:** Modulation of SEB-induced TSS by staphylococcal PAMPs.

HLA class II type	Treatment^a^	D-gal	Mortality
HLA-DR3	None	+	0/4
HLA-DR3	PGN	+	0/4
HLA-DR3	HKSA	+	0/4
HLA-DR3	SEB (10 *μ*g)	+	8/8
HLA-DR3	SEB (5 *μ*g)	+	6/6
HLA-DR3	SEB (10 *μ*g) + PGN	+	5/5
HLA-DR3	SEB (5 *μ*g) + PGN	+	4/4
HLA-DR3	SEB (10 *μ*g) + HKSA	+	3/3
HLA-DR3	SEB (5 *μ*g) + HKSA	+	4/4
HLA-DQ8	None	+	0/4
HLA-DQ8	PGN	+	0/4
HLA-DQ8	HKSA	+	0/4
HLA-DQ8	SEB	+	6/6
HLA-DQ8	SEB (10 *μ*g) + PGN	+	6/6
HLA-DQ8	SEB (10 *μ*g) + HKSA	+	6/6

HLA-DR3 and HLA-DQ8 transgenic mice were pretreated with D-galactosamine and an hour later challenged with the following agents. Animals were followed for 9 hours.

^a^Treatments given

PGN: peptidoglycan—50 *μ*g/mouse

HKSA: heat-killed* S. aureus*—10^8^ bacteria/mouse

SEB: staphylococcal enterotoxin B—10 *μ*g/mouse or 5 *μ*g/mouse

D-gal: D-galactosamine—30 mg/mouse 1 hour prior to being challenged with PGN, HKSA, SEB or at the same time.

## Abbreviations

PAMPs: Pathogen-associated molecular patterns
SSAg: Staphylococcal superantigen
PGN: Peptidoglycans
LTA: Lipoteichoic acid
HKSA: heat-killed* Staphylococcus aureus*
SEB: Staphylococcal enterotoxin B.
