# Supplementary material for: The Impact of Staphylococcus aureus-Associated Molecular Patterns on Staphylococcal Superantigen-Induced Toxic Shock Syndrome and Pneumonia
Source: Mediators Inflamm. 2014 Jun 12;2014:468285. doi: 10.1155/2014/468285 (PMC4082930; doi:10.1155/2014/468285)

Supplementary Fig 1. Modulation of expression of HLA-DR3 and CD86 on DC by HKSA. Splenic mononuclear cells from HLA-DR3 transgenic mice were cultured with medium, HKSA (10^8 bacteria/ml), SEB (1 μg/ml) or SEB+HKSA. 24 hours later, the cells were harvested, washed and expression of HLA-DR3 and CD86 on $CD11c^+$ was analyzed by flow cytometry. Charts depict mean fluorescent intensity (MFI). Each bar represents mean±SE from 2 different experiments.

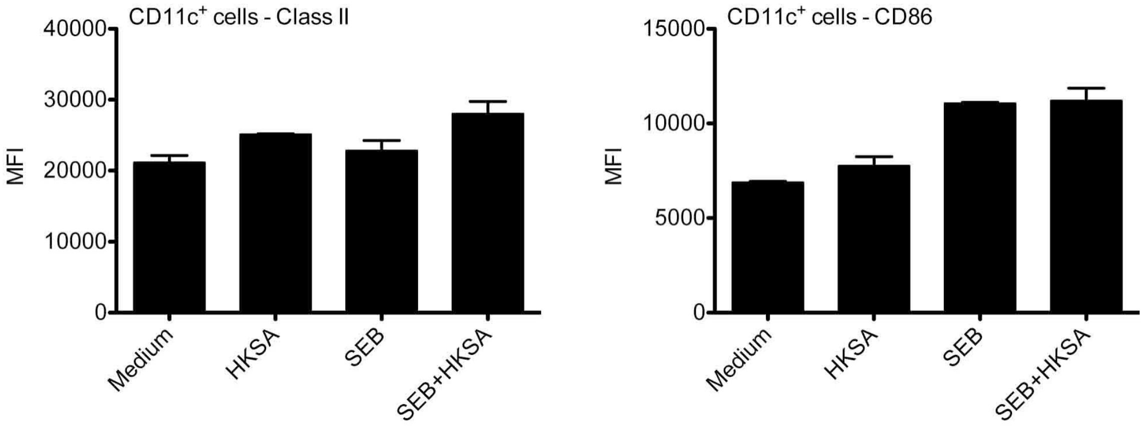

Supplement: Supplementary file 1 — Splenic mononuclear cells from HLA-DR3 transgenic mice were cultured with medium, HKSA (108 bacteria/ml), SEB (1μg/ml) or SEB+HKSA. 24 hours later, the cells were harvested, washed and expression of HLA-DR3 and CD86 on CD11c+ was analyzed by flow cytometry. Charts depict mean fluorescent intensity (MFI). Each bar represents mean ± SE from 2 different experiments. [file 468285.f1.pdf]
